# The Auditory Nerve Overlapped Waveform (ANOW) Detects Small Endolymphatic Manipulations That May Go Undetected by Conventional Measurements

**DOI:** 10.3389/fnins.2017.00405

**Published:** 2017-07-18

**Authors:** Jeffery T. Lichtenhan, Choongheon Lee, Farah Dubaybo, Kaitlyn A. Wenrich, Uzma S. Wilson

**Affiliations:** ^1^Department of Otolaryngology Washington University School of Medicine Saint Louis, MO, United States; ^2^Department of Communication Sciences and Disorders, Northwestern University Evanston, IL, United States

**Keywords:** electrocochleography, cochlear response, auditory nerve neurophonic, endolymphatic space, scala media, endolymphatic hydrops, Ménière's disease, cochlea

## Abstract

Electrocochleography (ECochG) has been used to assess Ménière's disease, a pathology associated with endolymphatic hydrops and low-frequency sensorineural hearing loss. However, the current ECochG techniques are limited for use at high-frequencies only (≥1 kHz) and cannot be used to assess and understand the low-frequency sensorineural hearing loss in ears with Ménière's disease. In the current study, we use a relatively new ECochG technique to make measurements that originate from afferent auditory nerve fibers in the apical half of the cochlear spiral to assess effects of endolymphatic hydrops in guinea pig ears. These measurements are made from the Auditory Nerve Overlapped Waveform (ANOW). Hydrops was induced with artificial endolymph injections, iontophoretically applied Ca^2+^ to endolymph, and exposure to 200 Hz tones. The manipulations used in this study were far smaller than those used in previous investigations on hydrops. In response to all hydropic manipulations, ANOW amplitude to moderate level stimuli was markedly reduced but conventional ECochG measurements of compound action potential thresholds were unaffected (i.e., a less than 2 dB threshold shift). Given the origin of the ANOW, changes in ANOW amplitude likely reflect acute volume disturbances accumulate in the distensible cochlear apex. These results suggest that the ANOW could be used to advance our ability to identify initial stages of dysfunction in ears with Ménière's disease before the pathology progresses to an extent that can be detected with conventional measures.

## Introduction

The Auditory Nerve Overlapped Waveform (ANOW) originates in the apical half of the cochlear spiral from afferent neural fibers tuned to low-frequencies (Lichtenhan et al., 2013, 2014, 2016). Conventional electrocochleography (ECochG) measurements such as the compound action potential (CAP) do not work adequately at low frequencies (Spoor and Eggermont, 1976; Picton, 2007; Sininger, 2007). The ANOW is derived from the cochlear response recorded from the auditory periphery. The cochlear response is an electrical measurement originating from the cochlear microphonic of inner and outer hair cells, changes to the lateral wall potential from slow or sustained current through hair cells, summating potential, excitatory postsynaptic potentials, CAPs from onset or phase locked neural excitation, and spontaneous excitation of single-auditory-nerve-fibers (Lichtenhan, 2012; Chertoff et al., 2015). When cochlear responses to alternating low frequency tones are averaged, the fundamental component and odd harmonics are canceled and the even harmonics are preserved. At low and moderate stimulus levels, the even harmonics originate from phase locked neural excitation. The result is a waveform with oscillation at twice the probe frequency. The ANOW technique advanced the work done with the auditory nerve neurophonic, which is simply a cochlear response evoked from low-frequency tones (Henry, 1995; Choudhury et al., 2012; Verschooten et al., 2012; Forgues et al., 2014; Verschooten and Joris, 2014; Koka et al., 2017). In particular, Lichtenhan et al. (2014) identified when the origin of the cochlear responses to low-frequency tones is, and is not, neural excitation from the apical cochlear half when stimulus level and recording location are varied.

The approaches used for the experiments reported here were three scala media manipulations that have been classically used to create, and study, endolymphatic hydrops. We found that the ANOW is considerably more sensitive to all of these manipulations than traditional objective measures of CAP thresholds and the endocochlear potential (EP): the amplitude of the ANOW was altered by each manipulation, while there were minimal changes to CAP thresholds or the EP. Hydrops induced by the small manipulations would not be accurately detectable by imaging techniques (Klis et al., 1990; Salt and DeMott, 1994a; Salt et al., 1995)–a consequence of fixative causing Reissner's membrane shrinkage and the transient nature of acute cochlear manipulations. These results suggest that measurements of ANOW amplitude have advantages over classically used measurements that are commonly used in the clinic and laboratory to identify and study endolymphatic hydrops.

## Materials and methods

### Surgical access of the endolymphatic space

To access the guinea pig endolymphatic space, the bony wall overlying the dark pigmentation of the stria vascularis was thinned with a flap knife and then an approximately 30 μm fenestra was made with a 1/3 mm House oval window pick (N1705 80, Bausch and Lomb Storz). Endocochlear potential (EP) measurements were used to verify the placement of the injection pipette into the endolymphatic space of the second cochlear turn. EP measurements used for experimental purposes were recorded from an additional fenestra in the third cochlear turn that accommodated an EP electrode. When a pipette was inserted into endolymph, there was no fluid leakage at the insertion site, suggesting that the site was effectively sealed. Experimental protocols for this study were approved by the Animal Studies Committee of Washington University School of Medicine in St. Louis (protocol numbers 20120113 and 20130069).

### Volume injection of artificial endolymph

Ears with chronic endolymphatic hydrops have an enlargement of the scala media cross sectional area. Injection of artificial endolymph can be used to model acute endolymphatic hydrops. Injections were made using double-barreled glass pipettes with tips beveled to an approximate 15–20 μm diameter. The injection barrel was filled with artificial endolymph (140-mM KCl and 25-mM KHCO3) while the second barrel was filled with 500 mM KCl and used to confirm placement in the endolymph with EP measurements. The pipette was mounted on a micro-syringe pump injector controlled with a micro-syringe pump controller (UMP3 and Micro4, respectively, World Precision Instruments). During the injections of artificial endolymph volumes into the second cochlear turn, EP measurements were made in the third cochlear turn. Injections of artificial endolymph were performed at rates of 5–10 nL/min for 15 min. The characteristic frequencies of our access sites to the second and third cochlear turns were estimated to be 2.5 kHz for second cochlear turn and 650 Hz for the third cochlear turn, based on the frequency-place map derived from guinea pig single auditory nerve fibers (Tsuji and Liberman, 1997) adjusted to the 20.8 mm length of the endolymphatic space.

### Iontophoretic Ca^2+^ delivery

Ears with chronic endolymphatic hydrops have been shown to have elevated endolymphatic Ca^2+^ (Ninoyu and Meyer zum Gottesberge, 1986; Meyer zum Gottesberge and Ninoyu, 1987; Salt and DeMott, 1994b, 1997; Fettiplace and Ricci, 2006). Administration of Ca^2+^ into the endolymphatic space can thus model some aspects of chronic endolymphatic hydrops. Ca^2+^ was iontophoresed into the endolymphatic space of the second cochlear turn using positive current. Pipettes for iontophoresis applications were made from single barreled glass with internal fiber. The pipette tip was beveled to a 2–3 μm diameter and filled with 160 mM CaCl_2_. The electrode tips were then filled with 0.5% agarose gel to prevent volume passive displacement of the electrolyte during the experiment (i.e., leakage of the electrolyte into the cochlea). The electrodes were stored with the tips in CaCl_2_ solution, allowing the electrolyte to equilibrate with the gel. Ca^2+^ was iontophoresed into endolymph for 15 min with 100 nA of current using a microiontophoresis current programmer (Model 260, World Precision Instruments).

### Tonal exposures

Brief exposures to low frequency tones at high, but non-damaging levels, (e.g., 95–115 dB SPL) have been shown to induce transient endolymphatic hydrops, the origin of the so-called “2-min bounce phenomenon” (Flock and Flock, 2000; Salt, 2004). We presented a 65 dB SPL 200 Hz tone at for 3 min in a closed acoustic assembly with a Sennheiser HD 265. During exposure to the tone, no sound-evoked potentials or acoustic emissions were collected.

### Statistics

Statistical analyses were completed in Statistical Analysis System 9.0 (SAS Institute, Cary, NC). A mixed model analysis with autoregressive covariance structure and cases as random factors was used to compare the change between the mean, pre-injection baseline measure (obtained between −10 and −1 min re. injection start) and the post-baseline measure (obtained between 0 and 30 min re. injection start) between the different tone burst frequencies and levels. Estimated marginal means and corresponding 95% confidence intervals were used to report the results of the interaction effect as well as the main effects in the mixed model. All statistical tests were two sided and evaluated at the alpha level of 0.01.

### Electrophysiological measurements

Tucker-Davis System 3 hardware controlled by custom-written software (Visual Basic, Microsoft) on a personal computer was used to make electrophysiologic measurements. A TD-RP2 was used to generate stimuli that were passed through TD-PA5 attenuators, and TD-HB7 headphone amplifiers. Cochlear responses were evoked from 50 tone bursts of alternating polarities. The duration of the tone bursts was 30 ms, and the duration of the linear two-cycle rise and fall times varied with stimulus frequency. Cochlear response measurements were made from 50 averages. An Etymotic ER-10C coupled to the hollow ear bar—a closed sound system—was used to deliver acoustic sound stimuli to the right ear of all guinea pigs. Calibrations were completed in individual ear canals by tracking 70 dB SPL tones from 0.125 to 26 kHz in ¼ octave steps.

Cochlear response measurements were made differentially between an Ag/AgCl ball-tipped electrode near the round window niche and a platinum-needle electrode near the vertex. An Ag/AgCl pellet electrode coupled to exposed soft tissue of the neck with a fluid bridge was used for grounding. ECochG measurements were made with a TD DB4 optically-coupled amplifier (1000x gain, 0.005–15 kHz bandpass filter) routed to an TD-RP2 module for digitization (48.8 kHz) and averaging. No artifact reject was applied.

**Figure 1:**
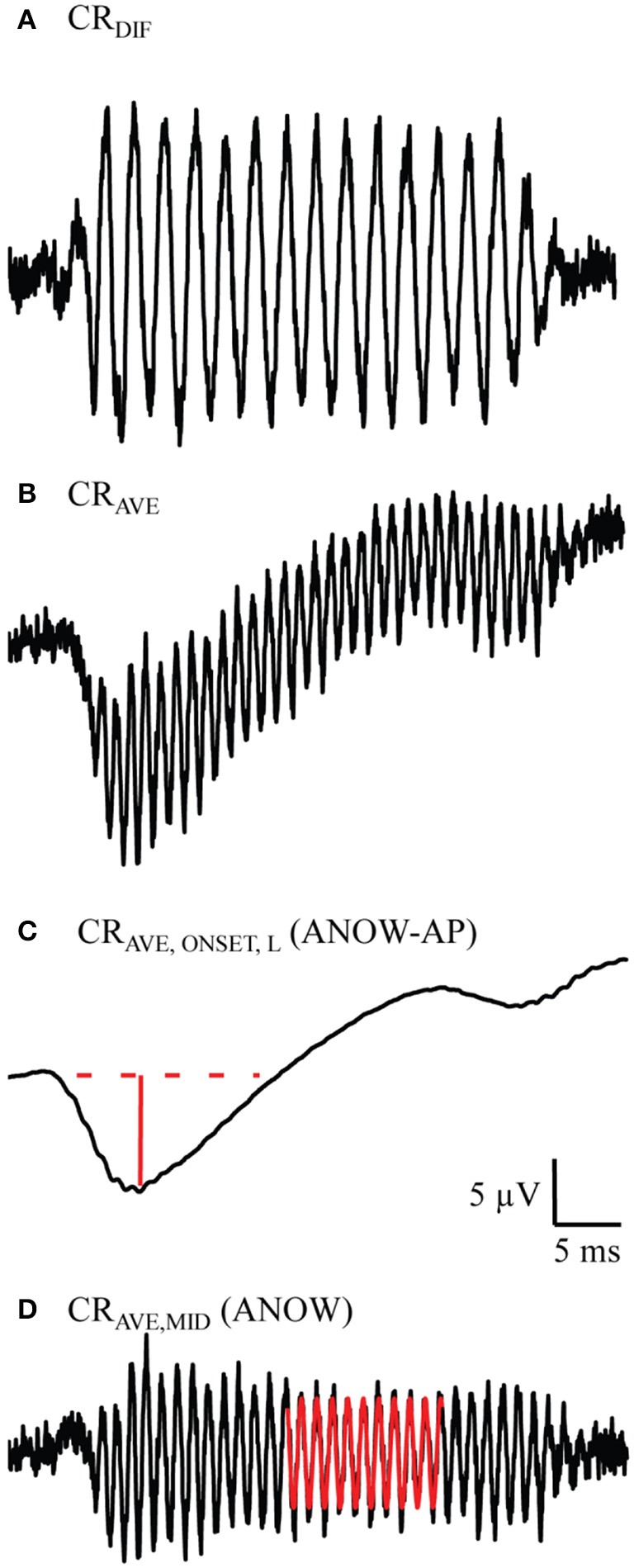
Example measurements used to measure the ANOW-AP and ANOW. These waveforms were evoked from 50 dB SPL 500 Hz tone bursts. Previous work demonstrated that the difference in cochlear responses to alternating tone bursts (CR_DIF_, **A**) is a blend of both hair cell and neural responses, but has been commonly referred to in the literature as the “cochlear microphonic” (Lichtenhan et al., 2014). Averaging cochlear responses to alternating tone bursts (CR_AVE_, **B**) and two-cycle smoothing (CR_AVE,ONSET,L_, **C**) yields the onset response to low-frequency stimuli. The amplitude of the ANOW-AP is the solid red line. Subtracting CR_AVE,ONSET,L_ from CR_AVE_ yields the CR_AVE,MID_ **(D)**, the waveform used to quantify the ANOW amplitude (red sinusoid).

Measurement names derive from terminology established in Lichtenhan et al. (2014). This terminology was based on stimulus conditions, not assumed cellular origins. The CAP is the commonly used waveform acquired from averaging responses to high-frequency tone bursts of alternating polarity, the CR_AVE,ONSET,H_. CAP thresholds were quantified with an automated procedure that identified the lowest stimulus level yielding a 10 μV N1 to P1 amplitude. The ANOW-AP is the CR_AVE,ONSET,L_, or the amplitude measurement of the two-cycle smoothed waveform (Figure 1C) from averaging cochlear responses to alternating polarity low-frequency tones (Figure 1B). The ANOW is the amplitude measured in the middle of the waveform resulting from averaging cochlear response to 500 Hz tone bursts of alternating polarity (CR_AVE,MID_, Figure 1D). The difference in cochlear responses to low frequency stimuli (CR_DIF_, Figure 1A) were not used in this study. Our previous work demonstrated that this waveform, which is commonly referred to as the “cochlear microphonic,” has cellular origins that vary with level and recording location (Lichtenhan et al., 2014), thus limiting the usefulness based on unsatisfactory interpretability.

## Results

### Volume injection of artificial endolymph

**Figure 2:**
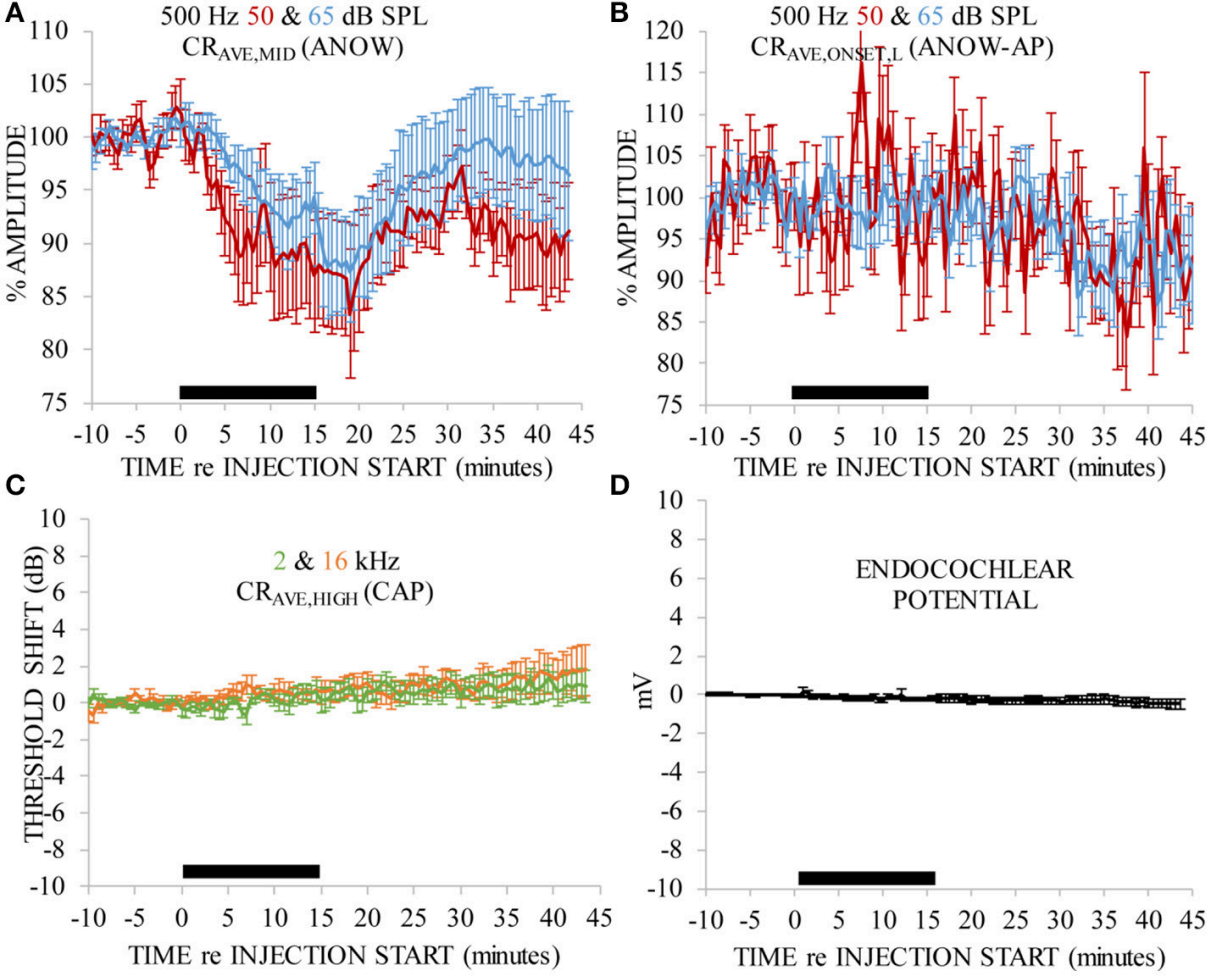
ANOW measurements were markedly affected by 75–225 nL of artificial endolymph injected into the second cochlear turn, but other measurements were not. Data in each panel were normalized to the average of 10 min of pre-injection measurements. Artificial endolymph was injected for 15 min, indicated by the thick black horizontal bar near the x-axis in each panel **(A–D)**. Error bars are standard errors of the mean calculated with measurements across all animals.

Volumes of artificial endolymph were injected into the second cochlear turn endolymphatic space at 5 (five ears), 10 (five ears), or 15 (three ears) nL/min for 15 min (75–225 nL total injection). Measurements made during each of these small, though different, injection rates are expressed together in these panels because each rate is very small, and produced similar effects, compared to classical use of artificial endolymph as a model of endolymphatic hydrops. In particular, the volume of artificial endolymph injected in our experiments were up to 16 times smaller than those used in previous contemporary experiments to create endolymphatic hydrops that was detectable by conventional CAP threshold measures (e.g., Sirjani et al., 2004). Laboratory norms for 500 Hz ANOW threshold is 45 dB SPL. Thus, ANOW to 50 dB SPL was 5 dB re. threshold. Volume injections caused significant reductions to the amplitude of the ANOW response to 50 and 65 dB SPL 500 Hz tone bursts between 0 and 30 min after the start of treatment (Figure 2A, degrees of freedom (df)(47), *p* < 0.01). Neural onset responses to both ANOW stimuli of either level (ANOW-AP, Figure 2B), the traditional CAP to threshold stimuli (Figure 2C), and EP measurements (Figure 2D) changed significantly (respectively, df(44), *p* < 0.01 df(47), *p* < 0.01, and df(10), *p* < 0.01). While statistically significant, we error on the side of caution and note that the changes to mean ANOW-AP were less than a mere 10%, CAP thresholds were less than 2 dB and EP changed less than 0.5 mV. Moreover, the reduction in ANOW amplitude were consistent and less variable than changes to other measurements. These results show that the amplitude of ANOW response can identify changes that may go undetected by conventional measurements.

### Iontophoretic Ca^2+^ delivery

**Figure 3:**
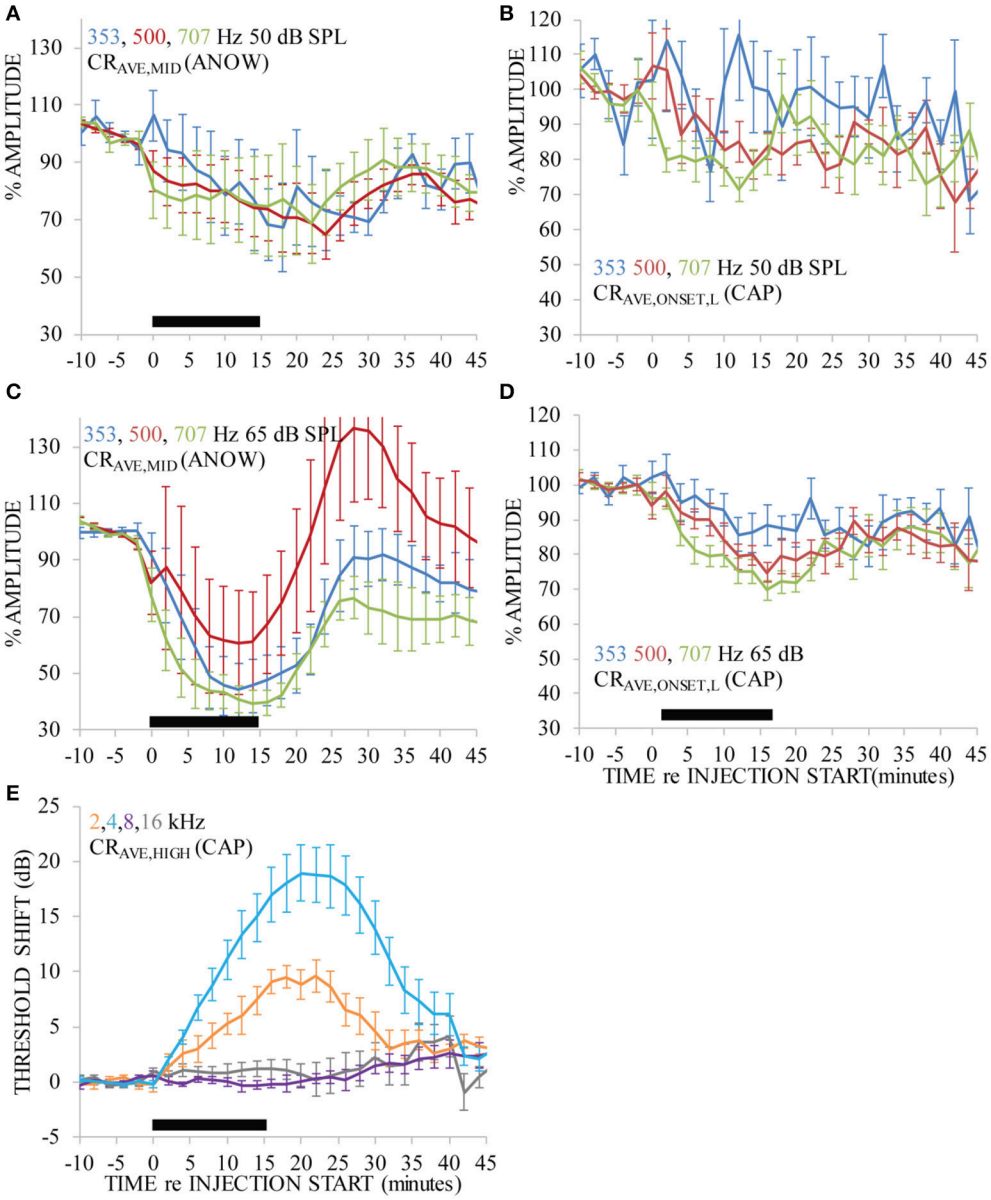
Ca^2+^ applied with 100 nA iontophoresis altered measurements that originate near the second cochlear turn administration site (i.e., CAPs to 2 and 4 kHz, **E**), as would be expected. But, ANOW-based measurements that originate in the apical cochlear half also changed significantly **(A–D)**, and CAP from cochlear regions distant to the administration site (i.e., 8 and 16 kHz) were not affected. Ca^2+^ was applied for 15 min, indicated by the thick black horizontal bar near the x-axis in each panel. Error bars indicate standard errors of the mean calculated with measurements across all guinea pigs.

Ca^2+^ was iontophoretically applied into endolymph with 100 nA current. Ca^2+^ was applied nine times to seven ears. That is to say, in two ears Ca^2+^ was applied twice: a second application was made after recovery from the first application. This treatment significantly affected the amplitude of the ANOW response to 50 dB SPL tone bursts of all selected frequencies [Figure 3A, df_(15)_, *p* < 0.01], as well as those to 65 dB SPL [Figure 3C, df_(33)_, *p* < 0.01]. ANOW onset responses (ANOW-AP) to 50 dB SPL stimuli were significantly affected [Figure 3B, df_(16)_, *p* < 0.01], as were ANOW-AP to 65 dB SPL [Figure 3D, df_(35)_, *p* < 0.01]. While the effect on ANOW-AP was significant, we note that the magnitude of the effect was to a lesser degree than the effect on ANOW (cf. Figures 3A,C). There were significant effects of time, frequency, and an interaction effect on CAP threshold [df_(36)_, *p* < 0.01; df_(48)_, *p* < 0.01; and df_(48)_, *p* < 0.01 respectively]. Pairwise comparison *post hoc* tests found that CAP thresholds to tone burst frequencies associated with cochlear frequency places nearest the administration site (i.e., 2 and 4 kHz) had a significant 10–20 dB threshold shift [Figure 3E; df_(64)_, *p* < 0.01 and df_(64)_, *p* < 0.01 respectively]. In contrast, CAPs to higher frequency stimuli associated with cochlear frequency places farther away from the administration site were not affected (i.e., 8 and 16 kHz). The close proximity of the second turn endolymphatic iontophoretic site to the spatial origin of 2 and 4 kHz CAPs, and the lack of CAP threshold change to 8 kHz, are consistent with a local and transient disturbance caused by the Ca^2+^ elevation.

It is remarkable that the amplitude of ANOW-based measurements was affected by the Ca^2+^ concentration that declines rapidly with distance from the iontophoretic site. The strong effect of Ca^2+^ on the amplitude of ANOW-based responses suggests it is either very sensitive to small Ca^2+^ disturbances or is sensitive to some other aspect of the Ca^2+^ manipulation, possibly including induced endolymph volume changes. Changes to the amplitude of ANOW-based measurements, but not to CAP thresholds measured away from the administration site, is consistent with volume disturbances in the apical half of the cochlea. Moreover, the amplitude of ANOW-based measurements to the higher 65 dB SPL stimuli detected the change more rapidly than traditional CAP threshold measurements at the same level, suggesting that higher stimulus levels were more sensitive to the induced physiological changes.

### 200 Hz exposures

**Figure 4:**
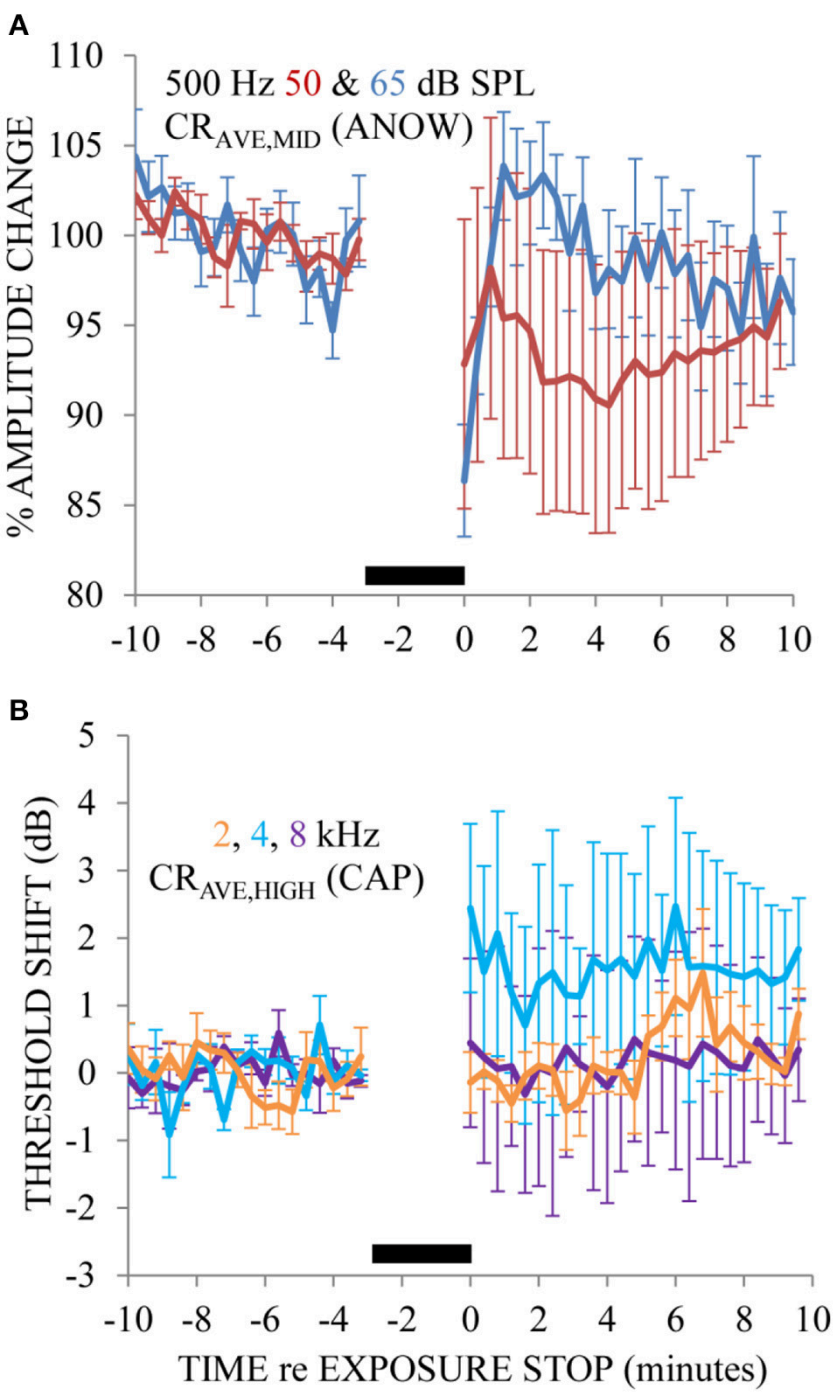
The ANOW amplitude to the 65 dB SPL stimulus level was affected most by 3 min of exposure to 65 dB SPL 200 Hz, and those to 50 dB SPL affected less so **(A)**. CAP thresholds to 2, 4, and 8 kHz were essentially unaffected by the endolymphatic hydrops induced by 200 Hz exposure **(B)**. No physiologic measurements were made during the 3 min of 200 Hz tone exposure, indicated by the thick black horizontal bar near the x-axis in each panel. Error bars are standard error of the mean estimates calculated with measurements across all animals.

Ears exposed to a 65 dB SPL 200 Hz tone caused the amplitude of the ANOW to “bounce” (i.e., rapidly decrease and then increase) and then slowly recover to pre-exposure measures (Figure 4A). These measurements were made from nine exposures to two ears. This effect happed over the first few minutes after the exposure stopped. Changes caused by 200 Hz exposure at this low level went undetected by traditional CAP threshold measurements that originate in the stiffer basal cochlear half to the extent that CAP thresholds did not bounce but the variability of these measurements increased (Figure 4B). The 65 dB SPL 200 Hz exposure used here is far less intense than the 115–120 dB SPL exposure that was required in previous experiments for the investigation of the “2-min bounce phenomena.” These measurements were made from nine exposures to two ears. Please note that we normalized the x-axis of Figure 4 differently than Figures 2, 3 so that the data could be more easily compared to previous reports on the 2-min bounce phenomena. The time course of the bounce in the amplitude of the ANOW to supra-threshold sounds is similar to that found in measurements originating in the less distensible cochlear base following a 115 dB SPL 200 Hz exposure that has been shown to cause endolymphatic hydrops in guinea pig ears (Salt, 2004). It is therefore possible that changes to ANOW could be caused by fluid volume disturbances in the distensible apical half of the cochlea.

## Discussion

### General

Traditional ECochG techniques do not effectively monitor diseased states in the low-frequency regions of the cochlear spiral. Indeed, it has been suggested that the lack of ECochG-based measures for low-frequency cochlear regions in normal and diseased ears is a likely origin of variable and discrepant findings throughout the literature (Palmer and Shackleton, 2009; Temchin and Ruggero, 2010). In the current study, we used the ANOW to study cochlear manipulations that have been previously used to simulate endolymphatic hydrops. We found that the ANOW could detect subtle dysfunction of the endolymphatic space that was missed by conventional CAP threshold measurements. The transient effects from our acute manipulations would not be detectable with time-intensive, traditional histological approaches used to measure the scala media cross sectional area.

Previous studies have concluded that the low-frequency hearing loss in ears with endolymphatic hydrops did not directly originate from the endolymphatic hydrops (e.g., Klis and Smoorenburg, 1988; Salt, 2004; Chihara et al., 2013), a possible consequence of fixatives causing Reissner's membrane shrinkage such that endolymphatic hydrops is underestimated and lacks correlation with physiologic measurements. But, these previous studies were limited to conventional physiologic measurements that work adequately for the basal cochlear half that is sensitive to high-frequencies. Our results are consistent with the hypothesis that low-frequency disturbances from endolymphatic hydrops would be greater for the more distensible cochlear apex that would partially close mechanoelectric transducer channels. The cochlear apex is one of the most distensible regions of the inner ear (Kimura and Schuknecht, 1965), a likely result of the gradation of basilar membrane width and stiffness. This gradation makes the apical cochlear half more prone to endolymph accumulation than other places along the cochlear length. Previously it was found that slow mechanical biasing of the cochlear partition with low-frequency tones had an effect that was inversely proportional with probe frequency (Lichtenhan, 2012). A related finding was that this “low-frequency biasing” was a sensitive indicator of sustained displacements of the organ of Corti, such as in ears with endolymphatic hydrops (Salt et al., 2009). Thus, it may be that low-frequency biasing the ANOW could be an even more powerful detector of hydrops than ANOW amplitude alone.

### Chronic endolymphatic hydrops and ecochg assessment

Two features of human Ménière's disease are endolymphatic hydrops and low-frequency sensorineural hearing loss. Endolymphatic hydrops is an enlargement of scala media due to accumulation of endolymph.

The relationship between measures of low-frequency hearing loss and endolymphatic hydrops is of interest because the origin(s) of low-frequency hearing loss in ears with Ménière's disease are still not known. The relationship between the severity of endolymphatic hydrops and low-frequency hearing loss is also not known. Various controversies and theories are fundamental to this interest. For example, (i) endolymphatic hydrops is not always associated with hearing loss in humans nor abnormal physiologic measurements in animals, yet human Ménière's diseased temporal bones with substantial hearing loss always have endolymphatic hydrops (e.g., Klis and Smoorenburg, 1988; Salt, 2004; Merchant et al., 2005; Nadol, 2010; Tagaya et al., 2011; Chihara et al., 2013; Yoshida et al., 2013). In other words, all Ménière's diseased ears have endolymphatic hydrops, but ears with endolymphatic hydrops do not always have symptoms of Meniere's disease. Presumably, there is a lag between the origins of endolymphatic hydrops and the low-frequency sensorineural hearing loss that is associated with Ménière's disease. (ii) A mere 10% of temporal bones from humans with hearing loss from Ménière's disease have sensory cell loss in the cochlear apex so the origin of the low-frequency loss is a mystery (Nadol, 2010). (iii) Acute endolymphatic hydrops increases the endocochlear potential, while chronic hydrops decreases the endocochlear potential (Meyer zum Gottesberge and Ninoyu, 1987; Salt, 2004). (iv) Since acute endolymphatic hydrops decreases the sensitivity of the operating point of the *in vivo* mechanics associated with the transfer of sound into excitement of neuronal membranes, but sustained cochlear partition displacements increase the sensitivity, it may be that mild endolymph accumulation during the early stage of Ménière's disease creates a feedback scenario which causes the diseased state to worsen (cf. Sirjani et al., 2004; Salt et al., 2009). (v) People with Ménière's disease often have word recognition scores that are worse than expected when considering their behavioral audiometric thresholds (Morrison, 1999). An ECochG-based approach to assessing low-frequency physiology in chronically diseased ears may therefore be helpful to address these controversies. ECochG can be minimally invasive and does not always require opening the cranium or cochlea, which could alter endolymphatic hydrops. *The results from our experiments reported here demonstrate that the conventional ECochG CAP thresholds were not as sensitive as ANOW amplitudes from supra-threshold stimuli to acute manipulations to the endolymphatic space. These findings suggest that ANOW may be useful for identifying initial stages of endolymphatic hydrops during the transition from early to late-stage chronic conditions when conventional measures of hearing would be unaffected*.

### ANOW assessment of acute models of endolymphatic hydrops

ANOW-based measurements, but not conventional CAP thresholds, changed in response to an acute induction of endolymphatic hydrops by injection of small volumes (5–15 nL/min for 15 min) of artificial endolymph to increase the volume of the endolymphatic space (Figure 2). The rates of volume injections used here were indeed small, as previous investigations induced endolymphatic hydrops with injection rates of 40–400 nL/min (Sirjani et al., 2004; Brown et al., 2013). The mechanism of endolymphatic hydrops from acute injections of artificial endolymph likely involves the reduction of mechanoelectric transducer current resulting from temporary volume and pressure increases that displace the organ of Corti toward scala tympani (Kakigi and Takeda, 1998; Wit et al., 2000), which is consistent with the recovery of ANOW measurements in Figure 2A. Chronic hydrops has traditionally been defined with visual detection of Reissner's membrane distension. But, reticular lamina distension toward the basilar membrane is a likely consequence of endolymphatic hydrops that would not be visible with traditional histological approaches. Changes to the organ of Corti height may occur, but could be misleading with histological measurements with fixatives that can cause hair cell contractions. In any event, a likely origin of the changes to ANOW response amplitudes changes we found could be slight, transient reticular lamina distention toward the basilar membrane, thereby changing hair cell function. A striking finding is that CAP thresholds to 2 kHz did not change while the ANOW measurements did indeed change (Figure 2). An injection of artificial endolymph into the second cochlear turn apically displaces the pre-existing volume at the injection site which flows through the 2 kHz region on the path to disrupting the cochlear apex (Salt and DeMott, 1997). This suggests the presence of demarcation region between 2 and 0.5 kHz that separates the stiffer basilar membrane in the basal half of the cochlea from the more distensible sensory structures in the apical half of the cochlea. This may be similar, or even related to, the region of basal-to-apical transition that identifies where cochlear mechanics are drastically different (e.g., Shera and Guinan, 2003; Abdala and Dhar, 2010, 2012; Shera et al., 2010; Temchin and Ruggero, 2010; Dhar et al., 2011; Moleti et al., 2017).

Chronic endolymphatic hydrops is associated with elevated endolymph Ca^2+^ levels, which most likely promotes closure of mechanoelectric transducer channels and contributes to endolymph accumulation (Ninoyu and Meyer zum Gottesberge, 1986; Meyer zum Gottesberge and Ninoyu, 1987; Salt and DeMott, 1994b, 1997; Fettiplace and Ricci, 2006). ANOW measurements changed dramatically in response to acute increases of endolymphatic Ca^2+^ levels, but conventional CAP measurements did not change (Figures 3B,D). The endocochlear potential rapidly drives Ca^2+^ out of scala media through non-selective cation channels that are located largely in hair cell stereocilia, as demonstrated by higher endolymphatic Ca^2+^ concentrations in the cochlear apex that has a 20 mV smaller endocochlear potential than in the base (Salt et al., 1989). Iontophoretic application of solutions to manipulate mechanoelectric transduction is a common procedure (e.g., Manley et al., 2004; Manley and Kirk, 2005; Sellick et al., 2006, 2007; Sellick, 2007). But, our approach and results are novel because the ANOW detected changes in response to small manipulations of the endolymphatic space.

The time course of ANOW changes from iontophoretically applied Ca^2+^ (Figure 3A) was faster than changes caused by volume injections of artificial endolymph (Figure 3C) presumably because Ca^2+^ instantaneously closes mechanoelectric transducer channels, rapidly affecting endolymph homeostasis. In contrast, slow injections of artificial endolymph in volume took longer to initiate flow from the injection site toward the cochlear apex. Maximal changes to 2 and 4 kHz CAP thresholds to iontophoretically applied Ca^2+^ were delayed compared to ANOW measurements to 65 dB SPL. Similarly, additional time was needed for iontophoretically applied Ca^2+^ to affect ANOW measurements to 65 dB SPL than to 50 dB SPL. These delays presumably originate from endolymph accumulation in the distensible apex that gradually affect the stiffer basal half of the cochlear spiral. Another issue regarding the time course of functional changes is that we suspect there are limitations to using volume injections and Ca^2+^ applications to model endolymphatic hydrops. We note that the function of some ears was likely deteriorating after 30 min of volume injection, likely contributing to a secondary decline in measurements in that time frame (cf. Figure 2).

Exposure to an intense, low-frequency tone for 3 min can initially cause a hearing threshold shift that is followed by a rapid recovery and finally a maximal shift that gradually recovers (Hirsh and Ward, 1952). The maximal shift occurs around 2 min after the tonal exposure stops, hence the name the “2-min bounce phenomena.” Salt (2004) demonstrated that endolymphatic hydrops was the origin of the bounce phenomena. Other findings related to this phenomenon are that the amplitude of reflection-source and distortion-product otoacoustic emissions can bounce (Kemp, 1986; Kirk and Patuzzi, 1997; Drexl et al., 2014, 2016), and that new spontaneous otoacoustic emissions can temporarily emerge from the noise floor (Kugler et al., 2014) but can be reduced in occurrence when the medial olivocochlear efferent system is activated (Kugler et al., 2015; Jeanson et al., 2016). A unique attribute of our findings is that the ANOW amplitude bounced to a low-frequency exposure tone having a sound pressure level as low as 65 dB SPL (Figure 4A), which was far less than the traditional exposures that were upwards of 115–120 dB SPL. The time course of the ANOW bounce was similar to bounces reported in other studies, suggesting the origin of the ANOW bounce was the same as that reported previously: endolymphatic hydrops. Bounces after exposure to a moderate-level, low-frequency tonal exposure supports our interpretation that the distensible cochlear apex where the ANOW originates is an ideal region for initial, or acute, hydrops.

## Conclusions

Until now, it has not been known which, if any, of the animal models of endolymphatic hydrops have disturbances in processing low-frequency sounds that would be consistent with the characteristic low-frequency dysfunction found in Ménière's disease. In the current study, we have found that the ANOW measurements from guinea pig, which originate from the auditory nerve fibers of the apical half of the cochlear spiral, are sensitive to manipulations of the endolymphatic space that are known to cause endolymphatic hydrops. ANOW changes were more sensitive than traditional CAP thresholds to the manipulations, suggesting that ANOW may be a useful technique to detect chronic endolymphatic hydrops in its initial stages.

## Author contributions

JL performed the experiments. JL, CL, KW, FD, and UW analyzed the data. JL wrote the manuscript.

### Conflict of interest statement

The authors declare that the research was conducted in the absence of any commercial or financial relationships that could be construed as a potential conflict of interest.
